# Supplementary material for: Examining the Efficacy of a ‘Feasible’ Nudge Intervention to Increase the Purchase of Vegetables by First Year University Students (17–19 Years of Age) in British Columbia: A Pilot Study
Source: Nutrients. 2019 Aug 2;11(8):1786. doi: 10.3390/nu11081786 (PMC6722732; doi:10.3390/nu11081786)
Supplement: Supplementary file 1 [file nutrients-11-01786-s001.pdf]

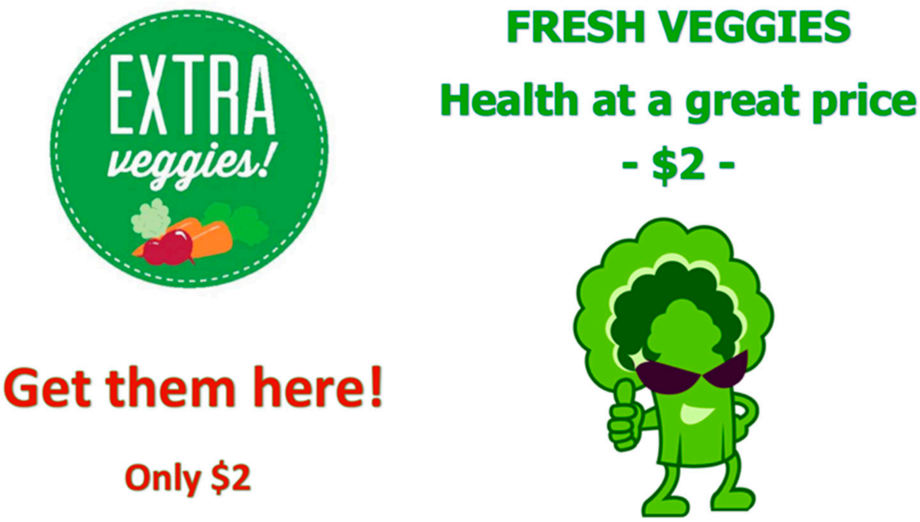

Figure S1. Specific signage images used as cognitive educational nudge.

Table S1. Breakdown in of numbers and percentage of cooked and fresh vegetables for the overall sample by phase.

	Baseline 1		Intervention 1		Baseline 2		Intervention 2	
Overall	N	%	N	%	N	%	N	%
No vegetables	3300	64.7	3865	62.6	3739	65.0	4791	64.9
Cooked Vegetables	1629	32.0	2034	32.9	1859	32.3	2345	31.8
Fresh Vegetable	169	3.3	274	4.4	156	2.7	249	3.4
Total Hot Table purchases	5098	100	6173	100	5754	100	7385	100

Table S2. Breakdown numbers and percentage of cooked and fresh vegetables for the female sample by phase.

	Baseline 1		Intervention 1		Baseline 2		Intervention 2	
Female	N	%	N	%	N	%	N	%
No vegetables	1334	65.5	1569	61.1	1611	65.1	1960	64.0
Cooked Vegetables	645	31.7	884	34.5	803	32.4	1013	33.1
Fresh Vegetable	58	2.8	113	4.4	62	2.5	90	2.9
Total Hot Table purchases	2037	100	2566	100,0	2476	100	3063	100

Table S3. Breakdown numbers and percentage of cooked and fresh vegetables for the male sample by phase.

Sex	Baseline 1		Intervention 1		Baseline 2		Intervention 2	
Male	N	%	N	%	N	%	N	%
No vegetables	1966	64.2	2296	63.7	2128	64.9	2831	65.5
Cooked Vegetables	984	32.1	1150	31.9	1056	32.2	1332	30.8
Fresh Vegetable	111	3.6	161	4.5	94	2.9	159	3.7
Total Hot Table purchases	3061	100	3607	100	3278	100	4322	100
